# Six gene and TH2 signature expression in endobronchial biopsies of participants with asthma

**DOI:** 10.1002/iid3.282

**Published:** 2020-01-05

**Authors:** Stephany Sánchez‐Ovando, Katherine J. Baines, Daniel Barker, Peter A. Wark, Jodie L. Simpson

**Affiliations:** ^1^ Faculty of Health and Medicine, Priority Research Centre for Healthy Lungs University of Newcastle New South Wales Australia; ^2^ Faculty of Health and Medicine University of Newcastle New South Wales Australia; ^3^ Respiratory and Sleep Medicine John Hunter Hospital New South Wales Australia

**Keywords:** asthma, endobronchial biopsy, inflammation, inflammatory phenotypes

## Abstract

**Background:**

Both the six gene signature (6GS: *CPA3, DNASE1L3, CLC, IL1B, ALPL*, and *CXCR2*) and T‐helper 2 signature (TH2S: *CLCA1, SERPINB2*, and *POSTN*) are proposed as biomarkers in the identification of inflammatory phenotypes of asthma in induced sputum and epithelial brushings, respectively. The aim of this study was to explore patterns of gene expression of known signatures, 6GS and TH2S in endobronchial biopsies.

**Methods:**

This was an exploratory cross‐sectional study of gene expression in endobronchial biopsies of 55 adults with asthma and 9 healthy controls (HC). The expression of the 6GS and TH2S was determined by quantitative polymerase chain reaction. Correlations with clinical and cellular characteristics were performed, and receiver operating characteristic was utilized to assess signatures' ability to predict asthma from HC and inflammatory phenotypes.

**Results:**

Gene expression of *DNASE1L3* (*P* = .045) was upregulated in asthma compared with HC, and *IL1B* (*P* = .017) was upregulated in neutrophilic asthma compared with non‐neutrophilic asthma. In asthma, the expression of *CPA3* was negatively associated with ICS daily dose (*r* = −.339; *P* = .011), *IL1B* expression was positively associated with bronchial lavage fluid (BLF) total cell count (*r* = .340; *P* = .013) and both *CLC* and *POSTN* expression were associated with lymphocytes percentage in BLF (*r* = −.355, *P* = .009; *r* = −.300, *P* = .025, respectively). Both 6GS (area under curve [AUC] = 86.3%; *P* = .017) and TH2S (AUC = 72.7%; *P* = .037) could significantly predict asthma from HC. In addition, 6GS can identify neutrophilic (AUC = 93.2%; *P* = .005) and TH2S identifies eosinophilic (AUC = 62.7%; *P* = .033) asthma.

**Conclusions and Clinical Relevance:**

There was increased expression of *DNASE1L3* in asthma and *IL1B* in neutrophilic asthma. These results show similar upregulated patterns of expression in two genes of the 6GS in endobronchial biopsies, previously identified in sputum. The upregulation of *DNASE1L3* and *IL1B* suggests that common mechanisms may be at play throughout the airway.

## Graphical abstract

Similar patterns of expression of known gene signatures were observed in endobronchial biopsies characterized by the upregulation of DNASE1L3 in asthma and IL1B in neutrophilic asthma suggesting these pathways are relevant in both biopsy and sputum in asthma.

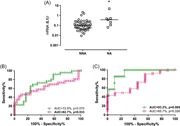

## 1 INTRODUCTION

Asthma is a heterogeneous disease with multiple clinical and inflammatory manifestations.^1^ Many genes and environmental factors play a role in pathogenesis,^2^, ^3^, ^4^ and many phenotypes have been described.^5^, ^6^, ^7^ The absence or presence of certain features determine the clinical manifestations, severity of asthma, and the response to treatment.^8^, ^9^ The first cases of asthma described were early onset, eosinophilic in nature, and related to allergic immune responses. More recently it is recognized that airway eosinophilia is not present in everyone with asthma and noneosinophilic phenotypes have been described.^10^, ^11^ In the majority of patients, asthma symptoms and exacerbations can be controlled by a combination of inhaled corticosteroids (ICS), acting to suppress the inflammation, and a β_2_ adrenergic agonist, acting to open the constricting bronchial smooth muscle. Although, corticosteroids have a pronounced effect in most patients, 30% to 40% of patients have a poor response to traditional treatment approaches and high sputum neutrophil counts have been linked to reduced responsiveness to corticosteroids as well as more severe asthma.^11^, ^12^ The identification of phenotypes of asthma based on the mechanisms of the disease pathogenesis that reflect clinical features offers the promise to better predict clinical outcomes and personalize response to treatment. Such an approach has utilized the identification of gene biomarker signatures that reflects pathogenic mechanisms associated with different airway inflammatory phenotypes of asthma. Previous transcriptomic studies of asthma have led to the identification of gene expression signatures including the six gene expression signature (6GS) in sputum samples^7^ and the T‐helper 2 signature (TH2S) in epithelial brushings.^2^ The 6GS comprises the expression of carboxypeptidase A3 (*CPA3*); deoxyribonuclease I‐like3 (*DNASEIL3*); Charcot‐Leyden crystal protein (*CLC*); alkaline phosphatase isozyme (*ALPL*); IL‐1β (*IL1B*); and chemokine (C‐X‐C motif) receptor 2 (*CXCR2*)^13^ to differentiate inflammatory phenotypes, corticosteroid treatment responsiveness, and future exacerbations.^7^, ^14^, ^15^ The TH2S comprises the expression of calcium‐activated chloride channel regulator 1 (*CLCA1*), plasminogen activator inhibitor 2 (*SERPINB2*), periostin (*POSTN*) for the identification of subjects with a TH2‐high immunity.^2^

Endobronchial biopsies sample structural cells including epithelial cells, mesenchymal cells, subepithelial matrix components as well as immune cells, which are not sampled in either sputum or epithelial brushings, but are involved in asthma pathogenesis. The aim of this study was to explore patterns of expression of known signatures, 6GS and TH2S in endobronchial biopsies of subjects with asthma compared with healthy, neutrophilic compared with non‐neutrophilic, and eosinophilic with noneosinophilic. We then explored associations between gene expression and clinical and cellular characteristics in participants with asthma, as well as the capacity of the signatures to predict disease status and inflammatory phenotypes.

## 2 METHODS

### 2.1 Participants

Sixty‐four adults recruited from the Respiratory Ambulatory Care Service, John Hunter Hospital, New South Wales, Australia. Adults (>18 years old) with no history of a clinical chest or upper respiratory tract infection in the previous 6 weeks were studied. Participants with asthma (n = 55) had a physician's diagnosis of asthma with previous confirmation of variable airway obstruction defined as either a post bronchodilator change in forced expiratory volume (FEV_1_) of at least 12% or 200 mL after 400 μg of salbutamol and/or the bronchial hyper‐responsiveness defined as at least 15% decline in FEV_1_ after inducing bronchial provocation with 4.5% saline solution. Bronchoscopy in healthy controls (HC) (n = 9) was conducted and none of the participants had underlying cardiac or lung disease. All HC had FEV_1_ percentage predicted more than 80% assessed by spirometry. Current smokers were excluded. Written consent was obtained from all the participating individuals. The study was approved by the Hunter New England Human Research Ethics Committee (05/08/10/3.09).

### 2.2 Study design

This is an exploratory cross‐sectional study in which clinical characteristics such as age, sex, body mass index (BMI), age of onset, asthma control questionnaire (ACQ) score, smoking history, bronchial lavage fluid (BLF) for cell count and endobronchial biopsies were collected during bronchoscopy.

### 2.3 Lung function

Spirometry was performed (Easy One Spirometer; Medical Technologies, MA) before bronchoscopy and without withholding usual medications on the day of the test. Percentage predicted for FEV_1_ and forced vital capacity (FVC) were calculated using The Third National Health and Nutrition Examination Survey (NHANES III).

### 2.4 BLF and endobronchial biopsies

Flexible bronchoscopy was performed to obtain BLF by wedging into the bronchus of the right middle lobe or lingula and lavaging with 20 to 40 mL of sterile normal saline. BLF was filtered and total cell count (TCC) and viability was measured.^16^ The BLF was centrifuged and the cell pellet was resuspended in phosphate‐buffered saline to the concentration of 1 × 10^6^/mL and cellular cytospins were prepared. The cytospins were stained with May‐Grünwald Giemsa (Beckman Coulter, Brea, CA) and differential cell count of 400 nonsquamous cells was performed. There were one to three endobronchial biopsies taken using alligator forceps from the second to third generation airways in the right or left lower lobe. Samples were stored in RNA later at −20°C.

### 2.5 Inflammatory phenotyping

Participants were characterized based on inflammatory phenotypes defined by the BLF cell count and differential determined from slides collected at the time of bronchoscopy. BLF cell count of HC (n = 82) from the cohort was utilized to determine a TTC cutoff for neutrophilic asthma (95th percentile = 0.99 × 10^6^/mL). Phenotypes were defined as follows: eosinophilic asthma as eosinophil greater than or equal to 3% of the TTC; noneosinophilic: less than 3% eosinophils; neutrophilic asthma: if neutrophils greater than or equal to 61% and greater than or equal to 0.99 × 10^6^/mL TTC, and non‐neutrophilic: less than 61% neutrophils of the TTC.^17^, ^18^, ^19^

### 2.6 RNA preparation

RNA was extracted from endobronchial biopsies using the RNeasy Mini Kit (Qiagen, Hilden, Germany). The quality of the RNA was assessed using Agilent 2100 Bioanalyzer (Agilent, Santa Clara, CA). RNA from samples had a mean (standard deviation [SD]) RNA integrity number value of 7.2 (SD 1.6).

### 2.7 Quantitative real‐time polymerase chain reaction

RNA (200 ng) from endobronchial biopsies was reverse‐transcribed to complementary DNA (cDNA) using the high‐capacity cDNA reverse transcription kit (Applied Biosystems, Foster City). Predesigned primers (*CPA3*: Hs00157019_m1; *DNASE1L3*: Hs00172840_m1; *CLC*: Hs00171342_m1; *ALPL*: Hs01029144_m1; *IL1B*: Hs01555410_m1; *CXCR2*: Hs01891184_s1; *CLCA1*: Hs00976287_m1; *SERPINB2*: Hs01010736_m1; *POSTN*: Hs01566750_m1; Applied Biosystems) were combined with Taqman gene expression master mix as per the manufacturer's instructions in duplicate singleplex real‐time PCRs (7500 Real‐Time PCR System; Applied Biosystems). Statistical analysis was performed using Δ*C*_T_ (cycle of threshold) of target gene subtracting the cycle threshold of housekeeping gene. Eukaryotic 18S ribosomal RNA with a *C*_t_ < 20 was considered acceptable. Fold change results were calculated by using $2^{-\Delta C_{t}}$ and scaled (×10^4^).^7^

### 2.8 Statistical analysis

Stata 15 (StataCorp, College Station, TX) was used to analyse data. Student *t* test was used for parametric data and Fishers' exact test for categorical data. For nonparametric data including gene expression we used the Wilcoxon rank‐sum test. Significance determined as *P* < .05. Data were reported in mean and SD for normally distributed and media and interquartile (IQR) Q1, Q3 for non‐normally distributed data. Spearman's rank correlation measured the association between gene expression and continuous measures of lung function and cell count in BLF. Logistic regression with receiver operating characteristic (ROC) curve analysis was utilized to evaluate gene set's capacity to identify asthma and inflammatory phenotypes.

## 3 RESULTS

### 3.1 Study participants

The characteristics of the participants are summarized in Table 1. Both groups were similar with respect to age, sex, and BMI. Lung function was significantly lower in participants with asthma compared with HC (FEV_1_% predicted mean (SD): asthma 77 (20.4), HC: 109.8 (13.5), *P* < .001; and FEV_1_/FVC mean (SD) asthma: 67.8 (12), HC: 81.4 (4.7); *P* = .001). The asthma cohort was comprised of predominantly females with adult onset of asthma, with 5 (9%) identified as former smokers with a median of 5 pack year. Total and differential cell count from the BLF are shown in Table 1. Participants with asthma had a significantly increased proportion of eosinophils in comparison to HC.

**Table 1:** Clinical and molecular characteristics of participants comparing asthma and healthy controls

Characteristic	HC	Asthma	*P* value
N	9	55	…
Age (y), mean (SD)	50 (14)	57 (15)	.193
Gender, F, n (%)	3 (33)	39 (71)	.028
Age of onset, n (%)			…
Childhood	N/A	20 (36)	
Adult	N/A	26 (47)	
Unknown	N/A	9 (16)	
FEV_1_% predicted, mean (SD)	109.8 (13.5)	77.0 (20.4)*	<.001
FEV_1_/FVC, mean (SD)	81.4 (4.7)	67.8 (12.0)*	.001
Former smoker, n (%)	1 (11)	5 (9)	.847
Pack years, median (IQR)	5 (5, 5)	5 (4.5, 5)	.617
BLF			
Total cell count ×10^6^/mL, median (IQR)	0.1 (0.1, 0.2)	0.1 (0.1, 0.6)	.324
Viability, median (IQR)	75 (60, 76)	78 (57, 91)	.505
Neutrophils %, median (IQR)	62.3 (15.3, 63.8)	47.3 (23.8, 78.8)	.481
Eosinophils %, median (IQR)	0.3 (0, 1.0)	2.5 (1.0, 8.0)*	<.001
Macrophages %, median (IQR)	23.5 (17.3, 40.5)	23.8 (12.8, 42.5)	.562
Lymphocytes %, median (IQR)	1.0 (0.3, 1.8)	0.5 (0, 2.0)	.643
Epithelial cells %, median (IQR)	14.5 (9.3, 20.0)	5.0 (1, 19.3)	.104
Squamous cells %, median (IQR)	2.4 (2.2, 4.3)	1.5 (0, 4.5)	.071
Endobronchial mRNA, median (IQR)			
6GS			
*CPA3*	0.63 (0.22, 1.71)	1.58 (0.51, 1.96)	.134
*DNASEIL3*	0.13 (0.06, 0.28)	0.22 (0.19, 0.25)*	.045
*CLC*	0.13 (0.06, 0.32)	0.07 (0.05, 0.08)	.144
*ALPL*	1.75 (0.75, 3.16)	1.46 (1.10, 1.86)	.628
*IL1B*	0.11 (0.05, 0.24)	0.17 (0.05, 0.19)	.754
*CXCR2*	0.18 (0.10, 0.42)	0.18 (0.07, 0.48)	.790
TH2S			
*CLCA1*	0.02 (0.01, 0.05)	0.03 (0.02, 0.07)	.171
*SERPINB2*	0.29 (0.13, 1.06)	0.19 (0.16, 0.36)	.550
*POSTN*	1.04 (0.43, 3.32)	0.61 (0.56, 0.89)	.124

*Note*: Data was analyzed using Wilcoxon rank or a Student *t* test depending on the outcome distribution and Fishers' exact test for categorical data.

Abbreviations: BLF, bronchial lavage fluid; FEV, forced expiratory volume; FVC, forced vital capacity; HC, healthy controls; IQR, interquartile range; mRNA, messenger RNA.

* Significant *P* ≥ .05 versus healthy controls.

### 3.2 Gene expression in asthma and HC

**Figure 1:**
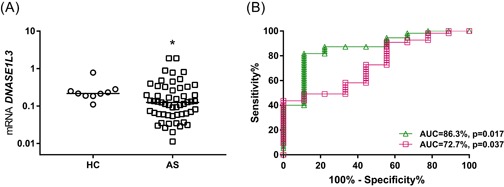
Gene signatures' performance in asthma. A, *DNASE1L3* upregulated in asthma compared with healthy controls (*P < .05); (B) ROC curves demonstrate that 6GS (green triangle/line) and TH2S (magenta square/line) can predict asthma from HC. AS, asthma; AUC, area under curve; HC, healthy controls; mRNA, messenger RNA; ROC, receiver operating characteristic

We assessed the expression of individual genes from the 6GS and TH2S signatures in asthma compared with HC. Participants with asthma had higher expression levels of *DNASEIL3* (*P* = .045) compared with HC (Figure 1A). There were no differences for the rest of individual genes in the 6GS and TH2 signatures between asthma and HC. There was no difference in the expression of the genes by sex (data not shown).

We performed ROC analysis to evaluate the signatures' ability to predict disease status in endobronchial biopsy. The AUC revealed that 6GS (AUC = 86.3%; *P* = .017) and TH2S (AUC = 72.7%; *P* = .037) could both significantly predict asthma from HC (Figure 1B).

### 3.3 Inflammatory phenotypes of asthma

Characteristics of asthma study participants with different inflammatory phenotypes are presented in Table 2. Participants with eosinophilic asthma (n = 26) were compared with noneosinophilic (n = 29) and also those with neutrophilic asthma (n = 7) were compared with non‐neutrophilic asthma (n = 48). Those with eosinophilic asthma had significantly lower BLF TTC and higher eosinophil percentage compared with noneosinophilic (Table 2). Those with neutrophilic asthma had a significantly lower ACQ score (*P* = 0.043) compared with non‐neutrophilic. Participants with neutrophilic asthma had significantly higher TTC, viability, percentages of neutrophils compared with non‐neutrophilic, while non‐neutrophilic had higher percentage of eosinophils, macrophages, epithelial cells, and squamous cell count in BLF compared with participants with neutrophilic asthma (Table 2).

**Table 2:** Characteristics of participants with eosinophilic and neutrophilic asthma

Characteristic	Eosinophilic	Noneosinophilic	*P* value	Neutrophilic	Non‐neutrophilic	*P* value
N	26	29	…	7	48	…
Age (y), mean (SD)	58 (14)	56 (16)	.478	62 (12)	56 (15)	.369
Gender, F, n (%)	16 (62)	23 (79)	.147	5 (71)	34 (71)	.974
BMI (kg/m^2^), mean (SD)	30 (7)	31 (8)	.882	24 (2)*	31 (7)	.017
Age of onset, n (%)			.109			.103
Childhood	7 (27)	13 (45)		4 (57)	16 (33)	
Adult	15 (58)	11 (38)		1 (14)	25 (52)	
Unknown	4 (15)	5 (17)		2 (29)	7 (15)	
FEV_1_% predicted, mean (SD)	77.2 (19.0)	76.8 (22.0)	.953	72.7 (21.3)	77.6 (20.4)	.558
FEV_1_/FVC, mean (SD)	67.8 (11.0)	67.7 (13.0)	.977	62.4 (11.8)	68.6 (11.9)	.209
Former smoker, n (%)	3 (12)	2 (7)	.357	0	5 (10)	.802
Pack years, media (IQR)	5 (4, 5)	5 (5, 5)	.564	…	5 (4, 5)	…
ACQ score, median (IQR)	2.3 (0.8, 2.8) (n = 21)	1.8 (0.9, 2.5) (n = 24)	.710	0.3 (0.3, 1) (n = 5)*	2 (1, 2.8) (n = 40)	.043
ICS use, yes n (%)	26 (100)	28 (96.6)	.596	7 (100)	47 (97.9)	.732
ICS dose mcg/d, median (IQR)	1300 (800, 2000)	2000 (800, 2000)	.117	2000 (2000, 2000)	1600 (800, 2000)	.100
GINA step 4‐5, n (%)	22 (85)	24 (83)	.473	6 (86)	40 (83)	.442
OCS use, yes, n (%)	3 (11.5)	2 (6.9)	.550	1 (14.3)	4 (8.3)	.609
OCS dose mcg/day, median (IQR)	10 (5, 10)	20 (15, 25)	.076	25 (25, 25)	10 (7.5, 12.5)	.147
BLF						
Total cell count, ×10^6^/mL, median (IQR)	0.11 (0.1, 0.3)**	0.2 (0.1, 1.1)	.032	6.4 (2.3, 6.9)*	0.1 (0.1, 0.4)	<.001
Viability, median (IQR)	68.8 (60, 84)	82 (50, 94)	.224	95.4 (94, 96.5)*	70.3 (50, 84.3)	<.001
Neutrophils %, median (IQR)	34.1 (24.5, 67.3)	59.5 (23.8, 84.5)	.158	89 (84, 91.5)*	39 (17.3, 67)	<.001
Eosinophils %, median (IQR)	8.1 (4.8, 20.8)**	1 (0.5, 2)	<.001	1.3 (0.5, 2.3)*	3.5 (1.1, 9.1)	.030
Macrophages %, median (IQR)	22.1 (14.5, 40)	29.8 (12.8, 48.3)	.312	8.8 (7.8, 13)*	27.6 (15.1, 44)	.003
Lymphocytes %, median (IQR)	0.5 (0, 1.3)	0.8 (0, 2)	.672	0 (0, 1)	0.8 (0, 2.3)	.154
Epithelial cells %, median (IQR)	7.5 (3.3, 24.8)	2.8 (0.8, 9)	.090	0 (0, 0.8)*	7.3 (2.5, 22)	<.001
Squamous cells %, median (IQR)	2 (0.3, 6.5)	0.7 (0, 3.2)	.115	0 (0, 0.3)*	2 (0.3, 5.2)	.006
6GS						
*CPA3*	0.7 (0.2, 1.7)	0.6 (0.2, 2.0)	.940	0.3 (0.1, 0.7)	0.7 (0.2, 1.9)	.225
*DNASEIL3*	0.1 (0.1, 0.3)	0.1 (0.1, 0.4)	.960	0.1 (0.1, 0.8)	0.1 (0.1, 0.2)	.419
*CLC*	0.14 (0.09, 0.38)	0.09 (0.04, 0.24)	.109	0.06 (0.05, 0.52)	0.13 (0.06, 0.31)	.495
*ALPL*	1.86 (1.07, 3.34)	1.73 (0.62, 2.97)	.469	1.73 (0.37, 5.88)	1.79 (0.79, 3.16)	.743
*IL1B*	0.10 (0.04, 0.24)	0.11 (0.06, 0.29)	.337	0.38 (0.11, 0.51)*	0.10 (0.04, 0.23)	.017
*CXCR2*	0.19 (0.09, 0.33)	0.18 (0.11, 0.46)	.578	0.20 (0.10, 0.46)	0.18 (0.10, 0.39)	.781
TH2S						
*CLCA1*	0.02 (0.01, 0.06)	0.02 (0.01, 0.03)	.363	0.01 (0.01, 0.04)	0.02 (0.01, 0.05)	.363
*SERPINB2*	0.51 (0.16, 1.88)	0.21 (0.09, 0.46)	.069	0.26 (0.05, 0.29)	0.31 (0.13, 1.29)	.251
*POSTN*	1.31 (0.56, 4.39)	0.70 (0.40, 1.95)	.183	0.70 (0.15, 2.99)	1.10 (0.43, 3.81)	.434

Abbreviations: ACQ, Asthma Control Questionnaire; BLF, bronchial lavage fluid; BMI, body mass index; GINA, Global Initiative for Asthma; ICS, inhaled corticosteroid; OCS, oral corticosteroid.

* Significant *P* ≥ .05 versus non‐neutrophilic.

** Significant *P* ≥  .05 versus noneosinophilic.

### 3.4 Gene signature expression in inflammatory phenotypes of asthma

**Figure 2:**
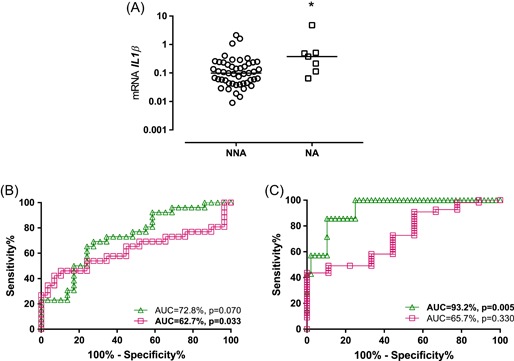
Gene signatures performance in inflammatory phenotypes of asthma. A, *IL1B* significantly upregulated in neutrophilic compared with non‐neutrophilic asthma (*P < .05). ROC curves demonstrate that (B) 6GS (green triangle/line) could not predict eosinophilic from noneosinophilic asthma, while TH2S (magenta square/line) showed capacity to predict; (C) 6GS but not the TH2S could predict neutrophilic from non‐neutrophilic asthma. AUC, area under curve; NA, neutrophilic asthma; NNA, non‐neutrophilic asthma; ROC, receiver operating characteristic

Gene expression for each of the individual genes from the gene signatures evaluated presented similar expression in eosinophilic asthma compared with noneosinophilic (Table 2). Gene expression for *IL1B* (*P* = .017) was significantly higher in those with neutrophilic asthma compared with non‐neutrophilic (Figure 2A).

ROC analysis revealed TH2S could predict eosinophilic from noneosinophilic asthma with a moderate discriminatory capacity (AUC = 62.7%; *P* = .033) (Figure 2B). Conversely, the 6GS but not the TH2S presented discriminatory capacity (AUC = 93.2%; *P* = .005) for neutrophilic from non‐neutrophilic asthma (Figure 2C).

### 3.5 Relationship of gene expression to clinical and cellular characteristics

We explored the associations between gene expression and clinical features presented in Table 1. Bronchial biopsy gene expression for *CPA3* was negatively associated with ICS daily dose (*r* = −.339; *P* = .011) suggesting higher expression when ICS doses are the lowest. Gene expression for *IL1B* was significantly and positively associated with BLF TTC (*r* = .340; *P* = .013), while both *CLC* and *POSTN* were significantly associated with BLF lymphocytes percentage (*r* = −.335; *P* = .009 and *r* = −.300; *P* = .025, respectively). There were no other associations found between expression of genes and clinical or inflammatory cell characteristics.

## 4 DISCUSSION

This study explored the expression of the gene signatures 6GS and TH2S previously reported in sputum and epithelial brushings in endobronchial.^2^, ^13^ We observed upregulation of two genes of the 6GS, an upregulation of *DNASE1L3* in asthma compared with HC, and upregulation of *IL1B* in neutrophilic asthma compared with non‐neutrophilic asthma. In addition, we evaluated the overall capacity of these signatures to identify disease status and inflammatory phenotypes, we observed that 6GS was able to predict asthma from HC, and neutrophilic from non‐neutrophilic asthma; and TH2S was able to predict asthma from HC, and eosinophilic from noneosinophilic asthma.

In an era of personalized medicine, the search and development of biomarkers that identify asthma and those that are more likely to benefit from a targeted therapeutic approach is an urgent unmet need. The majority of recent advances in asthma therapies have targeted TH2 mechanisms,^20^ however, with more than half of those with severe asthma exhibiting no evidence of active TH2 inflammation,^21^ there is a need to continue to explore the inflammatory profile and mechanisms in asthma. Our results support this importance, identifying genes not classically associated with a type 2 signature to be upregulated in the setting of treatment with ICS. Molecular phenotyping of well‐characterized people with asthma offers the hope that we will be able to identify and target new pathogenic mechanisms that will lead to novel therapies.^22^

*DNASE1L3* is an endonuclease that mediates the breakdown of DNA during apoptosis.^23^ Transcriptomic studies described it as an eosinophilic gene and responsive to ICS treatment in induced sputum of subjects with asthma.^7^, ^13^, ^24^ However, there is no further evidence that identifies specific roles of *DNASE1L3* in the pathogenesis of asthma. Our study showed upregulation of *DNASE1L3* in participants with asthma compared with healthy controls but no differences between inflammatory phenotypes of asthma. *DNASE1L3* is increased during apoptosis and plays an important role in fragmentation of DNA from the apoptotic vesicles; our results might be reflective of an overall increase of cellular apoptosis in participants with asthma.

When examining gene expression in neutrophilic compared with non‐neutrophilic, we observed a significant increase in *IL1B* in participants with neutrophilic asthma. Previous studies by our group have recognized *IL1B* gene expression associated with neutrophilic inflammation in induced sputum.^7^, ^13^, ^25^ It is well known that neutrophils are recruited from the proximal to the distal part of the airway to reside in the airway epithelium and submucosal glands, this process is mediated by IL‐8, IL‐1β, TNF‐α, and leukotriene B_4_.^26^ IL‐1β is produced mainly by macrophages, cultured bronchial epithelial cells, and neutrophils.^27^, ^28^ In those with asthma, the presence of neutrophilia has been associated with frequency of exacerbations,^28^ poor response to ICS,^29^, ^30^, ^31^ and disease severity.^32^ Simpson et al^33^ observed elevated expression of IL‐1β in subjects with neutrophilic asthma. It has also been reported that the inhibition of NLRP3 prevents neutrophilia and decreases airway hyper‐responsiveness.^34^

*CPA3* is a metalloexopeptidase specifically expressed in a particular subtype of mast cells in combination with tryptase.^35^ Expression of *CPA3* has been associated with TH2‐high asthma in sputum and epithelial brushings of steroid naïve asthma.^36^, ^37^ Berthon et al^15^ reported reduction of *CPA3* expression following treatment with oral corticosteroid, suggesting responsiveness to treatment. In addition, the number of mast cells containing tryptase and *CPA3* decreased following ICS treatment.^24^ In this study, we found a relationship between *CPA3* expression levels and ICS daily dose pattern that is consistent with what has previously been reported in induced sputum.

The discrepancies found in the expression of other genes investigated in this study and other studies and therefore, sample type, may be reflective of the compartmentalization of inflammation and variability of mast cells subtypes and eosinophils in the lung tissue compared with sputum and epithelial brushings.^38^ While application of gene signatures in biopsy samples is not a practical approach in distinguishing asthma from healthy controls and phenotypes, it may be helpful in determining common mechanisms with measureable activity in different compartments of the airways. ROC analysis demonstrated that 6GS when applied to endobronchial biopsies performed well in predicting asthma from HC, as well as neutrophilic from non‐neutrophilic asthma. Baines et al^7^ identified the 6GS by performing a transcriptomic analysis in induced sputum from a diverse group of subjects with asthma with varying treatment dose, clinical severity, and airway inflammatory phenotypes.^7^ This is quite similar to the population presented in this study. The TH2S was derived from epithelial brushings, so largely from a single structural cell type.^2^, ^5^ TH2S was found associated with eosinophilic airway inflammation, subepithelial basement membrane thickening, and response to ICS in a cohort with mild asthma who were steroid‐naïve.^2^, ^5^ This group was quite different from the cohort presented in this study comprised predominantly participants on high daily doses of ICS as described. Despite these differences we observed capacity of the TH2S to predict asthma from HC, and eosinophilic from noneosinophilic asthma.

A strength of this study is that it is the first to investigate these gene signatures in endobronchial biopsies in a well‐characterized cohort of adults with asthma and HC and to consider compartmentalization of the inflammation in asthma for mechanisms related to these signatures.^18^, ^39^ This exploratory study has particular limitations. Our study is cross‐sectional with a small sample size and does not consider different time points in participants with different inflammatory phenotypes and it is not clear how the expression of these genes may change over time or in response to known treatments. The discrepancies found in the expression of other genes investigated in this study compared with previous studies may be reflective of the compartmentalization of inflammation, variability of cells subtypes in the lung tissue compared with sputum and epithelial brushings.^38^ The correlations observed in this study are weak and a larger study is needed to explore complex interrelationships between gene expression and clinical features. The use of cell count cutoffs from induced sputum to identify eosinophilic and neutrophilic asthma in BLF is another limitation of this study. There is evidence that supports BLF eosinophils count resemble closely those found in induced sputum, however, the neutrophils cell counts are more variable and there is less agreement as to what should constitute a cutoff for neutrophilic asthma.^18^, ^40^ Furthermore, the HC group in this study presented high variability in percentage of BLF neutrophils. Studies on a larger cohort are needed to address these limitations.

In conclusion, our study sought to explore patterns of gene expression in endobronchial biopsies and to assess compartmentalization of inflammation in asthma by comparing our results to previously published studies. We have demonstrated upregulation of *DNASE1L3* in asthma compared with HC and *IL1B* in neutrophilic compared with non‐neutrophilic asthma. We confirmed the ability of 6GS to predict asthma from HC, and neutrophilic from non‐neutrophilic asthma in endobronchial biopsies, while the TH2S exhibited capacity to predict asthma from HC, as well as eosinophilic from noneosinophilic asthma. This study highlights the need for more research in larger cohorts to investigate further mechanisms of pathogenesis that may be reflected in endobronchial biopsies of subjects with different inflammatory phenotypes of asthma.

## CONFLICT OF INTERESTS

The authors declare that there are no conflict of interests.

## AUTHOR CONTRIBUTIONS

All authors designed the study. JLS supervised and coordinated the study. SS‐O performed the experiments and wrote the manuscript which was further refined and edited by JLS, PAW, KJB, and DB. PAW performed the bronchoscopy, KJB supervised the laboratory analysis, and DB supervised the statistical analysis. All authors approved the final manuscript for submission.

## Data Availability

The data of this study is available from the corresponding author upon request.
